# King Edward's Hospital Fund for London

**Published:** 1908-12-19

**Authors:** 


### KING EDWARD'S HOSPITAL FUND FOR LONDON.

### DISTRIBUTION MEETING.

A meeting of the President and General Council of King Edward's Hospital Fund for London, for the purpose of awarding grants to the hospitals, convalescent homes, and consumption sanatoria for the present year, was held on 14th inst. at Marlborough House, the Prince of Wales being in the chair.

Those present were: The Bishop of London, the Rev. Dr. T. Bowman Stephenson, Lord Bessborough, Lord Iveagh, Lord Rothschild, the Speaker, the Master of Elibank, M.P., the Postmaster-General, the President of the Hospital Sunday and Hospital Saturday Funds (the Lord Mayor), the Chairman of the County Council (Mr. R. A. Robinson), the Governor of the Bank of England (Mr. W. Middleton Campbell), the President of the Royal College of Physicians (Sir R. Douglas Powell), the President of the Royal College of Surgeons (Mr. Henry Morris), Sir Savile Crossley, Sir Joseph Dimsdale, Mr. Stuart Wortley, K.C., M.P., Sir Trevor Lawrence, Sir W. S. Church, Lieutenant-Colonel Sir Arthur Bigge, Sir Henry Burdett, Sir Everard Hambro, Sir Horace Marshall, Sir W. J. Collins, M.P., Sir John Craggs, Mr. J. Danvers Power, Dr. Edwin Freshfield, Mr. Frederick M. Fry, Mr. John G. Griffiths, Mr. William Latham, K.C., Mr. Albert G. Sandeman, Mr. Hugh C. Smith.

The minutes of the last meeting having been read and confirmed, letters of regret were reported from the following: The Lord Lieutenant of the County of London (the Duke of Fife), the Duke of Norfolk, the Chancellor of London University (Lord Rosebery), the Bishop of Southwark, Archbishop Bourne, the Rev. Dr. Guinness Rogers, the Chief Rabbi (the Rev. Dr. Adler), Lord Revelstoke, Lord Strathcona, Lord Farquhar, Sir Ernest Cassel, Mr. Burt, M.P., Sir John Aird, Sir Julius Wernher, Sir Edgar Speyer, Mr. J. Pierpont Morgan, jun., Professor W. Osler.

The following is the official report of the proceedings (which were held in private), to which we have added some headings to facilitate reference:

### The Income.

Lord Rothschild reported that the amount received by the Fund to December 10, after payment of expenses, was £156,748 1s. 5d.

Sir Henry Burdett stated that the total amount collected by the League of Mercy seemed likely to be larger than in any previous year, and that the grant to the King's Fund would be at least £18,000. The League had also made grants, amounting to £1,516, to sixty-eight hospitals in the Home Counties.

### Distribution Committee's Report.

The Chairman of the Distribution Committee (Sir William Church) read the report of that committee, as follows:—

1. The council having this year placed the sum of £135,000 in the hands of the committee for distribution amongst the London hospitals, as against £120,000 in 1907, the committee beg to report as follows:—

2. The number of hospitals applying for grants is 105, as against 99 last year, three having fallen out and nine new applications having been received. Of the new applications, four are from institutions which have become eligible for the first time as the result of the extension of the area from the seven to the nine mile radius from Charing Cross.

3. Among the hospitals which have fallen out is the North-West London Hospital, which has ceased to exist as a separate institution as the result of the decision to amalgamate with the Hampstead General Hospital. An increased maintenance grant of £1,500 becomes payable to the Hampstead General Hospital for five years on the completion of this amalgamation.

### Hospital Amalgamation.

4. In regard to the Throat, Nose, and Ear Hospitals, the Executive Committee in January last passed the following resolution:—

"The Executive Committee, while adhering to its policy in regard to the amalgamation of small hospitals, considers that the responsibility for the business of these amalgamations at present rests on the hospitals themselves, the King's Fund having by the sums provisionally set aside against the event of amalgamation shown its readiness to forward the desired object."

This resolution was communicated to the hospitals concerned, but, so far as the committee are aware, no step has been taken by them in the direction of amalgamation. The committee have set aside a further sum of £3,000 instead of making separate grants to each hospital. They cannot, however, recommend that this sum, together with the £3,000 already set on one side for the same purpose in the last two years, should be held over indefinitely, nor do they wish it to be understood that these amounts will be paid to the Throat and Ear Hospitals if the amalgamation is not carried out.

### Grants to Specified Objects.

5. In 1906 the council approved of a recommendation of this committee that grants made towards specified objects, such as building or other improvements, should in future be retained by the Fund until required, but that some limit should be placed on the length of time during which such grants should be allowed to remain unexpended, and that they should be reconsidered every two years while they remained unapplied. Of the grants made in December, 1906, and rated in accordance with this decision, the following amounts are still unpaid, namely:—

Dreadnought Hospital, £144 15s. 3d., being the balance of a grant of £2,000 to improvements.

Hospital for Women, £2,000 granted to rebuilding.

London Lock Hospital, £1,000 to nursing accommodation.

St. Mary's, Plaistow, £1,000 to building.

The committee recommend that the unpaid grants to the Dreadnought and to St. Mary's, Plaistow, be renewed, and that the grant to the Hospital for Women be renewed and transferred to the scheme of reconstruction which has now received the approval of the committee. In the case of the London Lock Hospital, the grant of £1,000 to nursing accommodation, which now lapses, was in addition to grants of £1,600—viz., £1,000 paid over in 1905 and £600 granted in 1907 and still retained by the Fund till required. The committee recommend that a sum of £4,000 should be added to the lapsed grant, and that the whole sum of £5,000 should be given to the rebuilding of the out-patient department at Dean Street (to which a substantial grant was promised in 1906 and 1907), on the understanding that the sum now needed to complete the fund for building the nurses' home at Harrow Road is subscribed by the public.

6. The sum of £5,000 voted towards the removal of King's College Hospital to South London increases the total amount contributed by the fund to this object to £27,000. The committee are glad to know that building operations on the new site are now in progress.

7. In February last the committee issued a circular setting forth the full purport and intention of the resolution of the general council that in future any new hospital, or those reconstructing or extending to a considerable extent within the area dealt with by the Fund, be requested, before taking definite action, to submit their proposals to the Fund. In the course of the year various schemes of greater or less magnitude have been submitted under this resolution. To some of these the committee have raised no objection, in some they have suggested modifications, while two or three are still under consideration. In some cases the result of the committee's deliberations upon these proposals is reflected in the grants recommended and in the accompanying public or private remarks.

The Question of Empty (?) Beds.

8. The committee have continued the policy of making substantial grants in aid of the extension of various hospitals situated in or near to the poorer districts remote from the larger general hospitals. In doing so they have not lost sight of the fact that in other parts of London there are beds or bed spaces unoccupied. They have carefully considered every case in which the existence of such vacant accommodation in institutions applying to the fund has been reported. The committee exclude from this category beds which are temporarily unoccupied merely as the result of the periodic changes of occupant, the provision of a surplus for accidental emergencies, and the occasional closing of wards for necessary cleaning. Apart from this inevitable margin, they find that most of the beds reported as vacant fall into the following classes:

(*a*) Beds which are at present used for the accommodation of nurses or for other necessary hospital purposes which could not be provided for elsewhere without expenditure on building;

(*b*) Beds, the accommodation for which was erected without due consideration of the local demand or of the prospect of maintenance, and which have never been opened for use;

(*c*) Beds which have recently become available as the result of extensions and which there is at present no reason to suppose the hospitals will not be able to open as public support (including the normal grants from this Fund) increases in response to the increased demand; and

(*d*) Beds in hospitals which have for one reason or another failed to secure or to retain public confidence.

The remaining beds which may properly be described as vacant for want of funds are less than 25, and in the case of some of these there are special circumstances which render their availability doubtful.

The committee consider that, in view of the existing distribution of the demand for hospital accommodation, the resources of the King's Fund can at present be better employed in assisting the extensions of the outlying hospitals above referred to than in opening the beds reported as vacant.

9. The reports of the visitors have, as heretofore, received the careful consideration of the committee and have been of the utmost value.

Uniform Balance-sheets.

10. The committee have found that the revised system of accounts, which provides for the publication of uniform balance-sheets and the separation of the expenditure upon the in-patient and the out-patient departments, has greatly facilitated the task of ascertaining the exact financial position of the various hospitals and of comparing their cost of working.

11. In addition to their published remarks, the committee have followed and extended their usual practice in recommending that various suggestions, including many arising from the visitors' reports, should be made privately to the hospitals. They have not omitted to call attention to the cases where, from the statistical report, it appears that greater attention to economical working is most urgently needed.

The details of the awards to the various hospitals are appended.

List of Hospital Awards.

Sir Savile Crossley presented the list of awards, as follows:—

The Distribution Committee desire to draw attention to the fact that the absence of a grant does not necessarily imply dissatisfaction.

In the following list the committee's remarks, where any are made, follow the amount of the grant in each case.

Alexandra Hospital for Children.—£500.

Belgrave Hospital.—£1,400; of which £1,000 to reduce building debt, on the understanding that a special effort is made to pay off the debt.

Blackheath and Charlton Cottage Hospital.—£150; of which £50 to overdraft and new lockers.

Bolingbroke Hospital.—£1,500.

British Lying-in Hospital.—£500.

Cancer Hospital.—The Committee are glad to see that no assistance from the fund is required.

Central London Ophthalmic Hospital.—£100; to equipment.

Central London Throat and Ear Hospital.—See paragraph 4 of report.

Charing Cross Hospital.—£3,500; of which £1,500 to overdraft.

Chelsea Hospital for Women.—£600; of which £100 to improvements.

Cheyne Hospital for Sick and Incurable Children.—The Committee are glad to see that no assistance from the Fund is required this year.

City of London Hospital for Diseases of the Chest, Victoria Park.—£3,000; increased grant, to assist in maintaining the 24 beds which the Committee are glad to see the hospital has recently opened.

City of London Lying-in Hospital.—£1,000; of which £500 to reduce debt.

Clapham Maternity Hospital.—£100.

Dreadnought Hospital.—£2,750.

Ealing Cottage Hospital.—£2,000; to rebuilding scheme. To be retained and paid over as wanted after the building scheme has been approved, and on the understanding that the new hospital will eventually be conducted on the lines of a metropolitan general hospital.

East End Mothers' Lying-in Home.—£750; of which £600 to reduce debt.

East London Hospital for Children.—£2,000; of which £1,000 to improvements, subject to a scheme being submitted and approved.

Eltham and Mottingham Cottage Hospital.—£100.

Evelina Hospital.—£1,300; of which £1,000 to reduce out-patients' building debt.

French Hospital.—£200.

Friedenheim Hospital.—£50.

General Lying-in Hospital.—£400; of which £100 towards surgical equipment.

German Hospital.—£200.

Great Northern Central Hospital.—£3,000; of which £1,000 to reduce debt.

Grosvenor Hospital for Women and Children.—£600; of which £250 for provision of mortuary.

Guy's Hospital.—£7,500; an exceptional grant was made in 1907.

Hampstead General Hospital.—£3,000; of which £1,500 to reduce debt; £1,500 maintenance grant as agreed upon amalgamation with the North-West London Hospital.

Home and Infirmary for Sick Children.—£250; of which £100 to improvements.

Home for Mothers and Babies, Woolwich.—£50; to equipment.

Hospital and Home for Incurable Children, Hampstead.—£25; in consideration of the fact that some curable cases are admitted.

Hospital for Consumption.—£3,500; the Committee view with special satisfaction the work done by this institution in its country sanatorium.

Hospital for Diseases of the Throat.—See paragraph 4 of report.

Hospital for Epilepsy and Paralysis.—£1,000; of which £500 to reduce debt.

Hospital for Invalid Gentlewomen.—£200.

Hospital for Sick Children.—£1,850.

Hospital for Women.—£4,000; of which £3,000 to the reconstruction, on condition that the whole sum required to complete the scheme is raised by October, 1909.

Hospital of St. John and St. Elizabeth.—£500.
Infants' Hospital.—The Committee are glad to see that no assistance from the Fund is required this year.
Italian Hospital.—£500.
King's College Hospital.—£8,000; of which £5,000 to Removal Fund and £1,000 to reduce debt.
Leyton, Walthamstow, and Wanstead Children's and General Hospital.—£250; of which £100 to reduce debt.
London Homœopathic Hospital.—£550.
London Hospital.—£12,000; of which £2,000 to reduce debt.
London Lock Hospital.—£4,500; of which £4,000 to the rebuilding of the out-patient department in Dean Street, on the understanding that the sum still needed for the nurses' home at Harrow Road is subscribed by the public (see paragraph 5 of report).
London Temperance Hospital.—£2,000; of which £1,000 to reduce debt.
London Throat Hospital.—See paragraph 4 of report.
Maternity Charity and District Nurses' Home, Plaistow.—£200; of which £100 to reduce debt.
Medical Mission Hospital, Balaam Street, Plaistow.—£300.
Memorial Cottage Hospital, Mildmay Park.—£50.
Metropolitan Hospital.—£2,500.
Middlesex Hospital.—£3,500.
Mildmay Mission Hospital.—£400; of which £50 towards surgical equipment.
Miller General Hospital for South-East London.—£2,000; of which £1,500 for extension on the same condition as last year—viz., that the present scheme is for a hospital of not more than 50 beds.
Mount Vernon Hospital for Consumption.—£1,500.
National Dental Hospital.—£75.
National Hospital for the Paralysed and Epileptic.—£3,000; of which £1,500 to the new building fund.
New Hospital for Women.—£1,000; of which £250 to improvements.
Norwood Cottage Hospital.—£150.
Paddington Green Children's Hospital.—£750.
Passmore Edwards Acton Cottage Hospital.—£50.
Passmore Edwards East Ham Hospital.—£50.
Passmore Edwards Hospital for Willesden.—£25.
Passmore Edwards Cottage Hospital, Wood Green.—£25.
Poplar Hospital.—£500.
Prince of Wales's General Hospital.—£3,000; of which £1,000 to reduce debt.
Queen Charlotte's Lying-in Hospital.—£750.
Queen's Hospital for Children.—£2,500; of which £1,000 to reduce debt, and £250 to removal of mortuary.
Royal Dental Hospital of London.—£1,500; of which £1,000 to special effort to reduce debt.
Royal Ear Hospital.—See paragraph 4 of report.
Royal Eye Hospital.—£1,250; of which £500 for improvements; the provision of a lift to be taken in hand first.
Royal Free Hospital.—£3,500; of which £1,500 to building debt.
Royal Hospital for Diseases of the Chest, City Road.—£750.
Royal Hospital, Richmond.—£250.
Royal London Ophthalmic Hospital.—£2,500.
Royal National Orthopædic Hospital.—£1,500; in accordance with amalgamation agreement.
Royal Waterloo Hospital for Children and Women.—£500.
Royal Westminster Ophthalmic Hospital.—The committee are glad to see that the hospital does not require assistance from the fund this year.
St. George's Hospital.—The committee are glad to see that owing to the large amount received from legacies the hospital is in no need of assistance from the fund this year.
St. John's Hospital for Diseases of the Skin.—£100.
St. John's Hospital, Lewisham.—£200.
St. Luke's House.—£50.
St. Mark's Hospital.—£100.
St. Mary's Hospital.—£4,000.
St. Mary's Hospital for Women and Children, Plaistow.—£1,500; of which £1,000 to building.
St. Monica's Hospital.—£100.
St. Peter's Hospital for Stone.—£100.
St. Saviour's Hospital, Osnaburgh Street.—£200.
Samaritan Free Hospital.—£1,000; of which £250 to reduce debt.
Santa Claus Home.—£25; in consideration of the fact that some curable cases are admitted.
University College Hospital.—£4,000; of which £1,000 to reduce debt.
Victoria Hospital for Children.—£1,200.
West End Hospital for Diseases of the Nervous System.—£1,250; of which £1,000 to rebuilding, subject to scheme being submitted and approved.
Western Ophthalmic Hospital.—£150; to reduce debt on the out-patient department.
West Ham and East London Hospital.—£2,000; of which £1,500 to building.
West London Hospital.—£4,500; of which £3,000 is an exceptional grant to reduce debt.
Westminster Hospital.—£2,000.
Wimbledon Cottage Hospital.—£75; of which £50 to improvements.
*Special Grant.*—Set aside for amalgamation of throat and ear hospitals ... ... ... ... £3,000

SUMMARY.

| | |
|---|---|
| Grants ... ... ... ... ... ... ... ... | £132,000 |
| Special Grant set aside for amalgamation of throat and ear hospitals ... ... ... | 3,000 |
| Total grants to hospitals for the year 1908 ... | £135,000 |

Convalescent Homes Report.

The Chairman of the Convalescent Homes Committee (Mr. William Latham, K.C.) read the report of that committee, as follows :—

1. Hitherto the Convalescent Homes Committee have dealt only with the sum of £1,000, which has for ten years been entrusted to the Fund annually by the trustees of the London Parochial Charities for distribution amongst convalescent homes. This year, however, the power of recommending as to distribution of grants not only to convalescent homes, but also to consumption sanatoria outside the metropolitan area, has been delegated to the committee as reconstituted under the Act of Incorporation. Applications from convalescent homes and consumption sanatoria were accordingly invited in the annual advertisements and Press notices; and the President and general council have placed in the hands of the committee the sum of £4,000 for both objects, in addition to the grant of £1,000 generously continued by the trustees of the London Parochial Charities for convalescent homes only. The committee beg to report as follows :—

2. The number of applications eligible for consideration amounted to 33 from convalescent homes and five from consumption sanatoria, as against 28 from convalescent homes last year.

3. The committee observe with regret the small number of consumption sanatoria which have applied for grants, and they have been impressed by the apparent absence of facilities for the transfer of consumption patients from London hospitals to country institutions at the time at which, in the opinion of the hospital doctors, they most need it. Two of the London consumption hospitals have their own country branches to which a certain number of patients can be so transferred. These branches are dealt with by the distribution committee in conjunction with the parent institutions. In the case of consumptive patients in other London hospitals there appears to be no means of securing the admission to sanatoria of those who specially need country treatment except by means of a subscriber's letter, which even after it is obtained involves a delay, often of many weeks, while the patient waits his turn for admission. The committee have made inquiries with a view to ascertaining whether this difficulty could be overcome, but have failed to arrive at any arrangement by which certain and prompt admission, in however small a number of cases, might be secured to London patients.

The committee note that it is not the practice of the Fund to accept subscribers' letters in return for grants, and they do not consider that this rule should be departed from in the case of country sanatoria. It is true that such letters would ensure that grants from the Fund would benefit London patients, but an addition to the number of letters issued by the sanatoria would only increase the delay experienced by the nominees of other subscribers without securing for a single patient in a London hospital the benefit of country treatment at the moment it is most needed.

4. As the small number of applications from suitable institutions may be partly due to the fact that the extension of the operations of the Fund to consumption sanatoria is a new departure which may not yet be generally known, the committee recommend that the sum of £1,000 out of the additional sum of £4,000 at their disposal this year should be carried for the present to a suspense account, and that in the event of any further communications being received the committee be authorised to make a supplementary recommendation for its distribution either to the annual meeting of the President and general council in the spring, or to the next ordinary distribution meeting in December, as the case may be.

5. In the circumstances set forth in the preceding paragraphs the committee have devoted a larger share of their total funds to convalescent homes than they would otherwise have done, and they would not desire it to be assumed that the grants to convalescent homes will necessarily be on the same scale in future years.

6. In some cases the committee have made grants in aid of building and other special purposes which they were unable to assist when they had only £1,000 of strictly income moneys to distribute. They recommend that such grants should be retained by the Fund until required, upon the same conditions as in the case of special grants to hospitals.

7. It has been the practice of the Fund in the case of hospitals in the London area not to assist in the foundation of new hospitals, nor to entertain applications for grants from hospitals which have not been in existence in a properly constituted form for a period of at least three years. But in view of the apparent lack of country accommodation for London consumptives the committee recommend that exceptions to this rule should at present be permitted in the case of consumption sanatoria in the country.

8. The committee are much indebted to the visitors who undertook the task of inspecting the consumption sanatoria in the country, and whose reports have been of the greatest value.

9. In addition to their published remarks, the committee have made suggestions privately to certain institutions. This practice has enabled them, amongst other things, to draw attention in some cases to the need for economy and to the fact that the revised uniform system of accounts applied to convalescent homes and consumption sanatoria as fully as to hospitals within the metropolitan area.

10. The grants to the various institutions are appended. The committee consider that the names of convalescent homes to which grants are not made should not be published. This has been their practice hitherto. In the case of consumption sanatoria, however, it seems to the committee desirable that the list should contain the names of all that have been admitted to consideration and inspected by the Fund's visitors.

### Convalescent Homes Awards.

Sir Savile Crossley presented the list of awards, as follows:

#### A.—CONSUMPTION SANATORIA.

The Convalescent Homes Committee desire to draw attention to the fact that the absence of a grant does not necessarily imply dissatisfaction.

In the following list the address of the institution follows its title, and the committee's "remarks" where any are made, follow the amount of the grant:

Children's Sanatorium, Holt, Norfolk.—The committee will be prepared to consider a grant when a scheme for a permanent sanatorium has been submitted and approved.

Eversfield Chest Hospital, St. Leonards.

National Association for the Establishment and Maintenance of Sanatoria for Workers, Benenden, Kent.—£1,000; to building.

Royal National Hospital for Consumption, Ventnor.—See paragraph 3 of report.

Royal National Sanatorium, Bournemouth.—See paragraph 3 of report.

#### B.—CONVALESCENT HOMES.

Alexandra Hospital for Children, Painswick.—£50.

All Saints' Convalescent Home for Children, Highgate.—£25.

Baldwin Brown Home for Convalescent Poor, Herne Bay.—£25.

Charing Cross Hospital, Limpsfield.—£300; of which £150 to reduce debt; £150 maintenance grant with a view to increasing the number of patients admitted free direct from the hospital.

Chelsea Hospital for Women, St. Leonards.—£50; with a view to increasing the number of patients admitted free direct from the hospital.

Convalescent Police Seaside Home, Hove.—£50.

East London Hospital for Children, Bognor, £250.

Home and Infirmary for Sick Children, Sydenham, Broadstairs.—£25.

Hospital for Sick Children, Great Ormond Street, Highgate. £250.

King's College Hospital, Hemel Hempstead.—£100.

London Hospital, Tankerton.—£100.

Mary Wardell Convalescent Home for Scalet Fever, Stanmore.—£25; to reduce debt.

Metropolitan Convalescent Institution (Children's Branch), Broadstairs.—£150.

Metropolitan Convalescent Institution, Walton.—£200.

Metropolitan Hospital, Cranbrook.—£50.

Middlesex Hospital, Clacton.—£400.

Mildmay Mission Hospital, Hendon.—£150; of which £100 to reduce debt.

Mrs. Gladstone's Free Convalescent Home, Mitcham.—£200; of which £100 to reduce debt.

Mrs. Kitto's Free Convalescent Home, Reigate.—£25.

National Hospital for the Paralysed, Finchley.—£50; with a view to facilitating the admission of patients without payment.

Paddington Green Children's Hospital, Slough.—£50.

Poplar Hospital, Walton-on-Naze.—£150; to building.

Queen Charlotte's Lying-in Hospital, Kilburn.—£25.

Surgical Home for Boys, Bantead.—£50; of which £25 to mortgage redemption fund.

Victoria Hospital for Children, Broadstairs.—£250.

| | |
|---|---|
| Grants to Consumption Sanatoria ... ... ... | £1,000 |
| Grants to Convalescent Homes ... ... ... | 3,000 |
| Grant to Consumption Sanatoria reserved ... ... | 1,000 |
| Total grants recommended ... ... ... | £5,000 |

### The Prince's Speech.

The Prince of Wales, in moving the adoption of the reports and awards, said:—My Lords and Gentlemen,—The recommendations contained in the reports of the Distribution and Convalescent Homes Committees, the adoption of which I have much pleasure in moving, will, I hope, be regarded with satisfaction, not only by the Council and by our subscribers, but by the public generally. (Hear, hear.) The donations for 1908 include the munificent gifts of £100,000 from Lord Mount-Stephen, which, in accordance with the direction of the donor, has been placed to capital, and £10,000 from Lord Iveagh, which has been similarly disposed of. Considerable sums have also been received from legacies, but it has been thought advisable to follow the established custom of distributing under this head an amount equal to the average receipts of past years. The sum thus available in the present year is £31,000. I am very glad to announce that the Fund has been in a position to allocate £140,000 for distribution among hospitals, convalescent homes, and country consumption sanatoria. (Hear, hear.) This is an increase of £19,000 over the sum distributed last year, and it is most encouraging to think that, in the past ten years, almost without any exception, there has been a steady increase in the annual amount available for distribution. Amongst the largest grants is that of £5,000 more to the removal fund of King's College Hospital, making a total of £27,000 granted by the Fund to this excellent object; and you will be glad to hear that the building on the new site at Denmark Hill has actually been commenced, so that adequate hospital accommodation will be provided in the near future in a district where it has long been needed. The grant of £12,000 to the London Hospital, including £2,000 towards the reduction of its debt, will be recognised as further evidence of the Fund's appreciation of the noble and efficient work carried out by that institution. The Fund continues to encourage the amalgamation of the smaller hospitals which are working on more or less parallel lines. It is hoped that the throat, nose, and ear hospitals may before long be willing to take action in this direction. The maintenance grants which would probably have been made to them in their separate existence have justly been reserved until the negotiations have assumed some more definite shape. It will be noticed from the Distribution Committee's report that the Fund adheres to the policy of aiding the extension of hospitals situated in or near to the poorer districts, and remote from the larger general hospitals, thus meeting the urgent needs of the dwellers in those localities. (Hear, hear.) The principle is one which involves a wide and detailed survey of the requirements of the Metropolis as a whole, and of the abundance or scarcity of hospital accommodation in its various parts. I observe with satisfaction that the committee have given the most careful consideration to this difficult question, and their report affords us much enlightenment and guidance in forming a judgment on the question of unoccupied beds. (Hear, hear.) We are again indebted to the London Parochial Charities for their annual grant of £1,000 for the convalescent homes, which we have been able to supplement this year. Those acquainted with the homes and the life of the London poor will agree that the use of convalescent homes to complete the cure begun in the London hospitals is, in many cases, most essential. I trust that the help of the London Parochial Charities may be continued, and that we may be able, year by year, to renew our efforts on behalf of these institutions. At our distribution meeting, a year ago, it was decided to extend the operations of the Fund to consumption sanatoria in the country, taking patients from London. Powers in this matter were delegated to the Convalescent Homes Committee, and you have just heard their report on this subject. It may be that this new departure of the Fund is not yet widely known, or that there are, as yet, only a small number of homes capable of dealing with what is an undoubted want. But, whatever the cause, the number of applications for grants from the Fund is unexpectedly small. It is common knowledge that the demands upon such well-known institutions as the Ventnor and Bournemouth sanatoria are very great. Admission to these is limited to patients who have obtained letters, and doubtless the rights thus assured to subscribers combine with the high reputation of the institutions to maintain their finances in their present creditable position. But even so, patients have almost invariably to wait for a considerable time for admission, and it is evident that these institutions meet only a portion of the existing demand. If the applications received by the Convalescent Homes Committee this year are to be taken as a measure of the supply of beds in the country for the consumptive poor of London, I cannot help feeling that the supply is inadequate. (Hear, hear.) One step towards meeting this need has already been taken by the formation of the "National Association for the Establishment and Maintenance of Sanatoria for Workers Suffering from Tuberculosis." The Benenden Sanatorium is the first outcome of this movement, and I think you will agree that such an effort on the part of the workers themselves to deal with this great question deserves the encouragement and support of the Fund. (Hear, hear.)

### More Annual Subscriptions.

I regret that the annual subscriptions do not increase as much as was contemplated by the originators of the Fund, especially as regards small amounts, and I hope that this will not be lost sight of by our committees; at the same time, we must not forget the liberal and steadily increasing contributions to the League of Mercy. Following upon the introduction of the revised uniform system of accounts, the statistical report which the Fund prepares every year upon the expenditure of the various hospitals has been considerably enlarged and extended. Eighteen more hospitals have been included, bringing the total number up to sixty-six, and the expenditure on in-patients has now, for the first time, been separated from that of out-patients. This has removed one possible source of error in the comparisons of cost per bed, and there is every reason to hope that the new series of reports, which has now been begun by Mr. Maynard, our secretary, will still further assist the control over expenditure which followed the initiation of the first series. Last year, in nominating members of committees, I stated that I proposed to proceed on the principle of not nominating any individual to more than one committee. This principle has worked satisfactorily during the past year, and for the ensuing year I do not propose to make any change in this respect. The fact that we have over a hundred members of the Fund, serving either as visitors or on this council, or on the various committees, is, I think, a guarantee to our subscribers, and to the general public, that the management of the Fund is widely distributed. The experience of last year suggested the necessity of certain amendments to the delegation resolutions agreed to in January last. Under my instructions these amendments have been drafted, and they lie on the table, together with a memorandum summarising these changes. If you should approve of the new form to-day, the meeting of the council, which it is necessary to hold early in the new year, need only be formal. I wish to take this opportunity of recording my sincere gratitude, and that of the council, to the honorary officials and members of the League of Mercy, numbering among them many of the keenest and most consistent supporters of the Fund from its inception, for the gratifying results of the past year, and for the continued increase of the yearly amount secured. To the honorary officials of the Fund I offer my warmest thanks for their devoted, untiring, and most valued services. Owing to the extension of the area of our operations, the work of the visitors has considerably increased, and I am deeply sensible of the careful and efficient manner in which it is done. I cannot conclude without referring to the lamented death of Mr. Vaughan Hawkins. His wise counsels and vast experience proved of the greatest benefit, both in the drafting of the King Edward's Hospital Fund for London Bill and in its guidance through the two Houses of Parliament. I have now much pleasure in moving the adoption of the reports. (Hear, hear.)

The Lord Mayor (Sir G. Wyatt Truscott) seconded the motion, which was supported by Mr. Danvers Power.

After discussion, the adoption of the reports was put and carried unanimously.

On the motion of Lord Iveagh, seconded by the Chairman of the London County Council (Mr. R. A. Robinson), it was resolved—That the cheques for the grants should be posted on December 17.

### Arrangements for 1909.

The President then said:—My Lords and Gentlemen,—With regard to the council for 1909, I have already alluded to our loss by the death of Mr. Vaughan Hawkins, and it is my intention to appoint the remaining members of the General Council of 1908 to serve again if they will kindly do so. I also desire to appoint the members of the Finance Committee who served last year. With regard to the Distribution Committee, I wish to appoint Sir William Church (chairman), Sir Trevor Lawrence, the Rev. Canon Barnett, Sir Henry Burdett, Sir Cooper Perry, Sir John Tweedy, Mr. Frederick M. Fry, and Mr. Oswald Partington. With regard to the Convalescent Homes Committee, I intend to appoint the same gentlemen as before, with the addition of Sir Horace Marshall and Sir Havelock Charles. With regard to the Executive Committee, I propose to appoint the same gentlemen as before, with the addition of Mr. Harry Lawson and Mr. William Whittall.

On the motion of the Governor of the Bank of England (Mr. W. Middleton Campbell), seconded by the Bishop of London, resolutions were approved delegating power to the Finance, Distribution, and Convalescent Homes Committees.

On the motion of Sir Everard Hambro, seconded by Sir Horace Marshall, a resolution was approved delegating powers to the Executive Committee.

On the motion of Mr. Stuart Wortley, seconded by the Master of Elibank, the council directed that it should be an instruction to the executive to report on the question of rules and regulations.

His Royal Highness then moved :—

"That the keys of the lock under which the corporate seal is kept be held as follows :—

"One of the three keys comprising one triplicate set shall be held respectively by the Earl of Bessborough, the Lord Rothschild, and Mr. Frederick M. Fry, and one of the three keys comprising the other triplicate set shall be held respectively by Sir Savile Crossley, Mr. J. Danvers Power, and Mr. Hugh Colin Smith."

### Vote of Thanks.

The Speaker of the House of Commons (the Right Hon. James W. Lowther, M.P.), in moving a vote of thanks to his Royal Highness for presiding, said that, the business before them being now concluded, he had the great honour of asking them to pass a vote of thanks to his Royal Highness for his kindness in taking the chair that day. He was sure it did not need any words from him, the youngest member of that council, to commend this to their attention, because it would receive the unanimous support of them all. The council was very much indebted to his Royal Highness for the great care and attention and time and study which he gave to the Fund. The poor of London and the cause of charity throughout the metropolis owed a great debt to his Royal Highness for having thrust himself so readily into this rather heavy work. As a representative of one of the Houses of Parliament, he would like to congratulate not only his Royal Highness, but himself, upon the fact that the intervention of Parliament did not seem to have inflicted any injury upon the cause, and that, as they had heard that day, the increased authority, the increased efficiency, and the growing usefulness of the Fund had not, at all events, been injured, and he thought they might flatter themselves that they had been advanced by the incorporation of the Fund under an Act of Parliament.

The Postmaster-General (the Right Hon. Sydney Buxton, M.P.), in seconding the vote, expressed the opinion that it was not so much the large sums of money distributed among the hospitals which had shown the value of the Fund as the conditions coupled with the grants. These had certainly brought about a very great improvement in hospital administration. The vote was carried by acclamation.

The Prince of Wales said,—Gentlemen, in thanking you for the vote of thanks which has been so kindly proposed by the Speaker, and seconded by the Postmaster-General, I am sure you would like to know that the King was much pleased to hear of the large increase in the sum which we have been able to distribute this year, and he has asked me to congratulate you on the excellent management of his Fund.

The proceedings then terminated.

